# Bleeding and thromboembolism due to drug-drug interactions with non-vitamin K antagonist oral anticoagulants—a Swedish, register-based cohort study in atrial fibrillation outpatients

**DOI:** 10.1007/s00228-020-03015-7

**Published:** 2020-10-07

**Authors:** Johan Holm, Buster Mannheimer, Rickard E Malmström, Erik Eliasson, Jonatan D Lindh

**Affiliations:** 1 Division of Clinical Pharmacology, Department of Laboratory Medicine, Karolinska Institutet, Stockholm, Sweden; 2 Clinical Pharmacology, Karolinska University Hospital, Stockholm, Sweden; 3 Department of Clinical Science and Education at Södersjukhuset, Karolinska Institutet, Stockholm, Sweden; 4 Internal Medicine, Södersjukhuset, Stockholm, Sweden; 5 Department of Medicine Solna, Karolinska Institutet, Stockholm, Sweden

**Keywords:** Anticoagulants, Drug interactions, Hemostasis, Pharmacokinetics, Thrombosis

## Abstract

**Purpose:**

To study the association between interacting drugs and bleeding or thromboembolism in atrial fibrillation outpatients treated with non-vitamin K antagonist oral anticoagulants (NOACs).

**Methods:**

Population-based cohort study of outpatients treated with NOACs in Sweden from 2008 to 2017. Patients with atrial fibrillation and newly initiated NOAC treatment were identified in the Prescribed Drug Register. Comorbidities and outcome data were retrieved from the Patient Register and the Cause of Death Register. Cox-regression analyses were performed to evaluate the primary endpoints any severe bleed and ischemic stroke/transient ischemic attack/stroke unspecified during the first six months of treatment. Secondary endpoints were gastrointestinal bleeding, intracranial bleeding, ischemic stroke, and venous thromboembolism.

**Results:**

Increased risk of any severe bleed was found when NOAC treatment, and drugs with pharmacodynamic effect on bleeding were combined, compared to NOAC only. An increased risk with these combinations was evident for apixaban (hazard ratio (HR) 1.47; 95% CI 1.33–1.63), rivaroxaban (HR 1.7; 95% CI 1.49–1.92), and dabigatran (HR 1.26; 95% CI 1.05–1.52). For apixaban, there was an increased risk of any severe bleed when combined with CYP3A4 and/or P-glycoprotein (P-gp) inhibitors (HR 1.23; 95% CI 1.01–1.5). The use of inducers of CYP3A4 and/or P-gp was low in this cohort, and effects on ischemic stroke/TIA/stroke unspecified could not be established.

**Conclusion:**

Increased risk of bleeding was seen for pharmacodynamic and pharmacokinetic interactions with NOACs. Prescribers need to be vigilant of the effect of interacting drugs on the risk profile of patients treated with NOACs.

**Electronic supplementary material:**

The online version of this article (10.1007/s00228-020-03015-7) contains supplementary material, which is available to authorized users.

## Introduction

Non-vitamin K antagonist oral anticoagulants (NOACs) have become increasingly utilized in Sweden and the rest of the world during the past two decades, and the drug group is the first-line treatment for stroke prevention in atrial fibrillation [1–4]. Apixaban is more commonly chosen than the other NOACs, whereas rivaroxaban, dabigatran, and edoxaban, in descending order of use, are also increasingly used among Swedish patients [2]. Patients risk formation of intracardiac thrombi as a consequence of atrial fibrillation, leading to systemic embolism and ischemic stroke, or hemorrhages due to treatment with anticoagulants. The risk-benefit profile of NOACs has proved superior to warfarin or no treatment [1, 5, 6]. However, multimorbidity and polypharmacy add to the risks requiring attention in the treatment of patients with atrial fibrillation. Hypertension, heart failure, diabetes, stroke, and myocardial infarction were the most common comorbidities in this patient group in the pivotal NOAC trials [7]. Furthermore, polypharmacy is frequent, in the ARISTOTLE trial (apixaban) in atrial fibrillation patients, the median number of concomitantly used drugs was 6, and 76.5% of the patients fulfilled the definition of polypharmacy (5 or more drugs) [8]. Similarly, in the ROCKET-AF trial (rivaroxaban), the median number of concomitant medications was 5, and 64% of patients were exposed to polypharmacy [9]. Generally, population-based studies have shown that a higher number of drugs are associated with an increased exposure to potential drug-drug interactions, and associations between polypharmacy and an increased risk of major bleeding or stroke have been implicated in NOAC patients from pivotal studies [10–12].

In general, NOAC drug interactions may arise by two principally different mechanisms. Pharmacodynamic interactions result from concomitant treatment with drugs with individual effects on hemostasis or risk of bleeding. Pharmacokinetic interactions are caused by impact of other drugs on the systemic level of NOACs [1]. P-glycoprotein (P-gp) is a critical drug transporter expressed both in the liver, in the intestinal wall, and in the kidney. This efflux pump promotes lowering of systemic levels of its transporter substrates including all NOACs. In addition, cytochrome P 450 (CYP) 3A4 has an important role in metabolic clearance of apixaban and rivaroxaban in the liver and the intestinal wall [13]. Consequentially, drugs that inhibit or induce P-gp may affect exposure to all NOACs, whereas interactions via CYP3A4 are primarily expected for apixaban and rivaroxaban. Guidelines and recommendations on potential drug interactions with NOACs are primarily based on pharmacokinetic studies, and there are few studies on the overall clinical effects of potential drug interactions with NOACs [12, 14, 15].

The aim of this study was to evaluate whether concomitant treatment with NOACs and interacting drugs was associated with increased risks of bleeding or thromboembolic events in patients with atrial fibrillation in Swedish outpatient care, compared to the use of NOAC only.

## Methods

### Study design and data sources

This study was a retrospective cohort study, based on data from three national Swedish registers, the Prescribed Drug Register, the Patient Register, and the Cause of Death Register [16]. Patients with atrial fibrillation that had been prescribed any NOAC since the introduction of these drugs in Sweden were included in the cohort. Composite primary endpoints were any severe bleed (gastrointestinal bleeding, hemorrhagic stroke, other intracranial bleeding, other severe bleeding) and ischemic stroke/stroke unspecified/TIA, for potential bleeding and thromboembolism interactions, respectively. Secondary endpoints were selected components of the primary endpoints, gastrointestinal bleeding, intracranial bleeding, and ischemic stroke and the separate endpoint venous thrombosis. The Prescribed Drug Register contains information on all dispensed prescription drugs in Sweden. All drugs prescribed in outpatient health care or drugs taken from drug storage rooms in nursing homes are included in the register, but not over the counter (OTC) drugs or drugs used in hospital inpatient care. Consequentially, outpatient prescription drug use in the country is very well covered [17]. Data retrieved from the Prescribed Drug Register included the code according to the Anatomical Therapeutic Chemical (ATC) classification system of the drug, dispensation date, and the size of the dispensed prescription in defined daily doses (DDDs) [18]. The Patient Register contains information on all healthcare visits in specialized outpatient and inpatient care. Information on diagnosis according to the International Classification of Diseases (ICD), procedure codes, and dates for diagnosis are available for each care event [19]. The Cause of Death Register contains information on the time of all deaths that occur in Sweden, with ICD diagnoses specifying the cause of death and contributing causes. The above registers are maintained by the National Board of Health and Welfare and data can be linked between registers using the Swedish personal identity number, unique to each individual.

### Study cohort

For patients that had been prescribed any NOAC (apixaban, rivaroxaban, dabigatran or edoxaban) between 2008 and 2017, data on all prescribed drugs were retrieved from the Prescribed Drug Register between 2007 and 2017. In addition, data from the Patient Register between 1998 and 2017, and the Cause of Death Register between 2008 and 2017, on diagnoses and procedure codes (Online Resource, Suppl. Table 1), and time of deaths, were retrieved for these patients. The first NOAC was introduced in Sweden in 2008, and 2017 was the last year for which data were available in all registers. Patients with atrial fibrillation and a new initiation of NOAC treatment were included in the study. Indications for NOAC treatment in Sweden include atrial fibrillation, deep venous thrombosis and pulmonary embolism, and postoperative prophylaxis following hip or knee replacement. The Patient Register does not fully cover primary health care, where some patients receive atrial fibrillation diagnoses. In contrast, diagnoses of other indications for NOAC treatment are given mainly in specialized health care. To include patients with presumed atrial fibrillation in the study, patients with diagnosis of any of the other indications within 60 days of initiated NOAC treatment were excluded. Furthermore, patients with mechanical valve or mitral stenosis were excluded. A washout period of 90 days before NOAC treatment was implemented for vitamin K receptor antagonists and previous NOAC treatment.

### Definitions of exposure

Treatment periods for NOAC and interacting drugs were identified through linkage of drug dispensations. Drug treatment was identified based on the ATC-code/s of each substance (Online Resource, Suppl. Table 2). Linked treatment periods were estimated based on the number of DDDs of consecutive dispensations. A dispensation that occurred within the time frame defined by 2.5 x the number of DDDs of the previous dispensation, but less than 100 days after the previous dispensation, was considered a linked continuous treatment. Since the DDD does not always account for the actual prescribed dose, multiplying with 2.5 allowed for a halved dosing regimen to be linked. However, a limit of 100 days prevented overestimation of linkage between dispensations. This limit was set based on the circumstance that patients on long-term treatment are dispensed drugs every third to fourth month, a consequence of the Swedish reimbursement model.

Drugs that potentially interact with NOACs were identified in the Janusmed interactions database [20] and in guidelines from the European Heart Rhythm Association (EHRA) [1]. Clinically relevant interactions defined as contraindicated, or where dose adjustment is recommended (class D or C, respectively, in Janusmed interactions) in any of these two sources, were identified. Potentially interacting drugs were classified according to the mechanism of interaction with each NOAC, into drugs with pharmacodynamic effect on bleeding, CYP3A4 and/or P-gp inhibitors, or CYP3A4 and/or P-gp inducers (Online Resource, Suppl. Table 3). Additionally, drugs that were listed as having a pharmacodynamic effect on bleeding with any NOAC were included for all NOACs in the study. The first episode with a new initiation of NOAC treatment for each patient was included in the analysis. For patients exposed to more than one NOAC, the first treatment episode for each NOAC was included. Potentially interacting concomitant drug treatment initiated before or at the time of initiation of the NOAC defined exposure to a potential interaction.

Descriptive data is presented for baseline variables with proportions or mean values and standard deviations. For all descriptive baseline variables and baseline covariables used in the analyses, three separate time frames were defined to identify ICD diagnoses before the index date. Consequentially, diagnoses within 10 years, 5 years, or 6 months before index date were included depending on clinical relevance (Online Resource, Suppl. Table 1). Patients were followed up for 6 months after initiation of NOAC treatment. Censoring occurred at the end of exposure to either NOAC or the interacting combination or death. Migration was assumed to result in the end of exposure based on the Swedish register data.

### Statistical analyses

Cox-regression survival analyses were performed for all endpoints. Outcome events were defined as receiving either a primary or secondary diagnosis for the outcome in the Patient Register or the Cause of Death register during the time of exposure (Online Resource, Suppl. Table 1). The hazard ratio for bleeding events or thromboembolic events was analyzed for apixaban, rivaroxaban, and dabigatran with or without interacting drugs presumed to potentiate the risk according to the Janusmed interactions database or EHRA guidelines. Patients exposed to edoxaban were too few in the dataset to allow analysis. All analyses were adjusted for the mechanistic interaction groups not currently analyzed. Analyses of risk of bleeding was adjusted for selected components of HAS-BLED [21, 22] (hypertension, renal disease, liver disease, ischemic stroke/stroke unspecified/TIA, any severe bleed, anemia, age category, and alcohol abuse). PK-INR was not available. Additionally, the use of platelet aggregation inhibitors was included in the mechanistic group with pharmacodynamic effect. The analyses of risk of thromboembolism were adjusted for the components of CHA_2_DS_2_-VASc (heart failure, hypertension, diabetes, ischemic stroke/TIA/arterial embolus/stroke unspecified, vascular disease, age category, and sex) [22, 23]. Crude outcome free survival probabilities of the primary outcomes were presented in Kaplan-Meier curves.

All variables were categorical in the analyses. To evaluate the proportional hazards assumption, we analyzed Schoenfeld residuals and performed exploratory Cox-regression analyses with covariate-time interactions. In addition, analyses to evaluate statistical interaction between covariates were conducted. We retrieved data on all patients fulfilling inclusion criteria from the Prescribed Drug Register. However, power analyses indicated 83–89% probability of successfully identifying a hazard ratio (HR) of 2 with 1000 patients exposed to an interaction, depending on the NOAC analyzed.

All statistical analyses and managing of the datasets were performed using R version 3.6.1 [24]. Statistical significance was defined at the 5% level (two-sided). HRs are presented with 95% confidence intervals (CI).

## Results

**Fig. 1:**
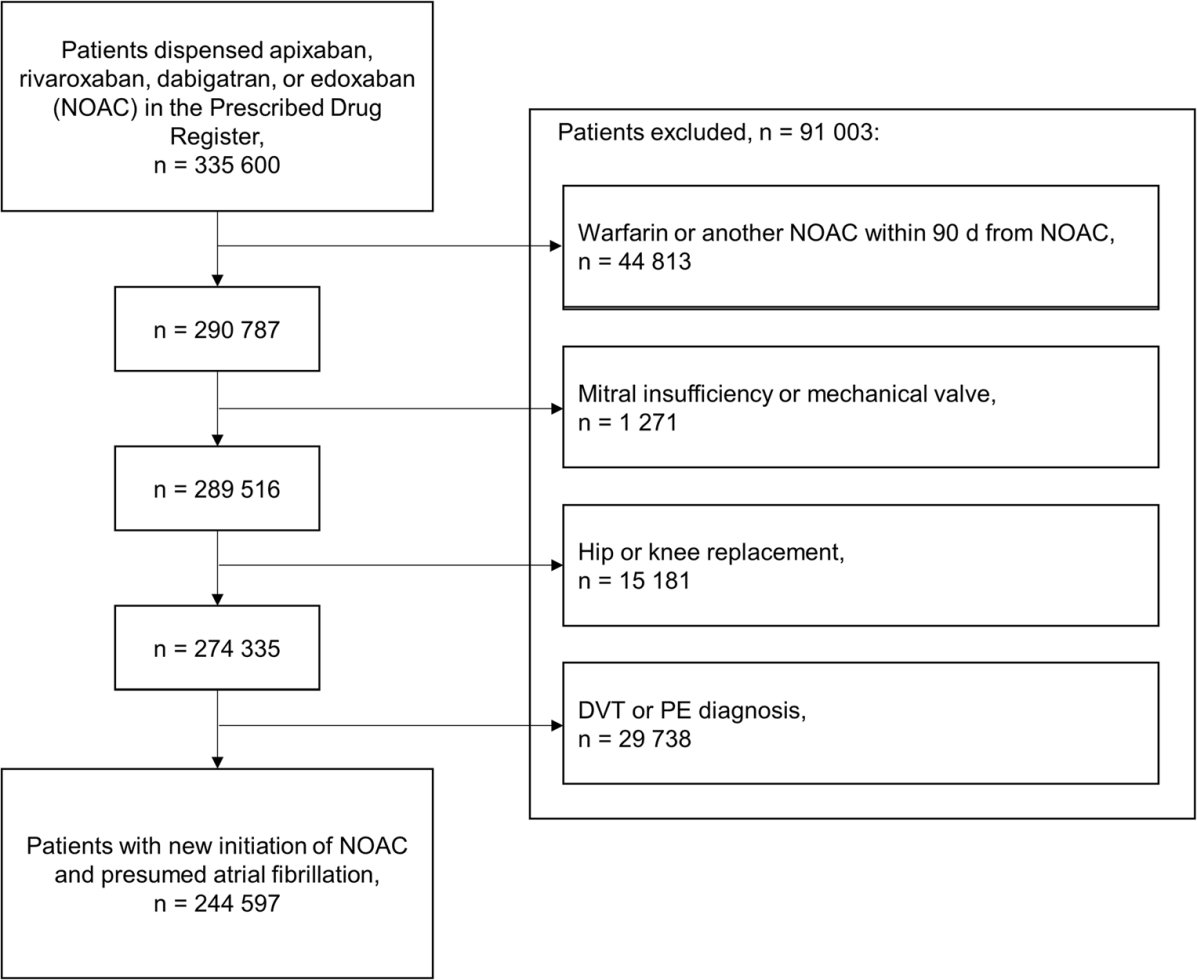
Study inclusion flow chart of 244,597 patients with newly initiated NOAC treatment during 2008–2017 in Swedish outpatient care. Excluded groups of patients are shown in the order of exclusion from the dataset

Among outpatients dispensed NOACs in Sweden during the study period, from 2008 to 2017, 244,597 patients with presumed atrial fibrillation had new episodes of NOAC treatment (Fig. 1). The percentages of patients with apixaban, rivaroxaban, and dabigatran were 61, 24, and 15% (the percentage of patients with edoxaban was less than 1%) (Table 1). Potential interactions with pharmacodynamic effect on bleeding were found in 48% of the patients, whereas inhibitors and inducers of CYP3A4 and/or P-gp were less common, 4 and 1%, respectively. More than half of the patients exposed to inhibitors or inducers of CYP3A4 and/or P-gp also had drugs with pharmacodynamic effect on bleeding, whereas only a few percent of patients with pharmacodynamic interactions had drugs from the pharmacokinetic groups. The distribution of age and sex was similar between patients without potential interactions and patients with the respective interaction groups. Patients without potential interactions had less cardiovascular morbidity, the mean CHA_2_DS_2_-VASc score was lower, and previous bleeding events were less common at baseline. Additionally, other comorbidities, e.g. cancer, diabetes, and renal disease, were more common among patients exposed to potential interactions.

**Table 1:** Baseline characteristics for 244,597 patients with newly initiated NOAC treatment during 2008–2017 in Swedish outpatient care. Values are given as *n* (%) or mean (± SD)

	NOAC *n* = 244,597	No interaction *n* = 122,476	Pharmacodynamic effect *n* = 116,499	CYP3A4 and/or P-gp inhibitor *n* = 8927	CYP3A4 and/or P-gp inducer *n* = 3314
NOAC					
Apixaban (%)	148,108 (61)	74,808 (61)	69,564 (60)	5841 (65)	2136 (64)
Rivaroxaban (%)	59,276 (24)	29,505 (24)	28,597 (25)	1894 (21)	783 (24)
Dabigatran (%)	35,617 (15)	17,524 (14)	17,475 (15)	984 (11)	375 (11)
Edoxaban (%)	1596 (< 1)	639 (< 1)	863 (< 1)	208 (2)	20 (< 1)
Interacting drug groups					
Pharmacodynamic effect (%)	116,499 (48)	0 (0)	116,499 (100)	4596 (51)	1974 (60)
CYP3A4 and/or P-gp inhibitor (%)	8927 (4)	0 (0)	4596 (4)	8927 (100)	126 (4)
CYP3A4 and/or P-gp inducer (%)	3314 (1)	0 (0)	1974 (2)	126 (1)	3314 (100)
Age and sex					
Age, mean (SD)	72.1 (12.9)	71.1 (13.3)	73.2 (12.4)	72.4 (11.4)	71.9 (12.3)
Age < 65 years (%)	56,057 (23)	30,948 (25)	23,666 (20)	1938 (22)	753 (23)
Age 65–74 years (%)	76,467 (31)	39,263 (32)	35,402 (30)	2959 (33)	1040 (31)
Age 75–79 years (%)	39,560 (16)	18,948 (15)	19,678 (17)	1570 (18)	595 (18)
Age 80+ years (%)	72,513 (30)	33,317 (27)	37,753 (32)	2460 (28)	926 (28)
Female (%)	114,740 (47)	56,811 (46)	55,624 (48)	3582 (40)	1490 (45)
Cardiovascular morbidity					
CHA2DS2VASc, mean (SD)	2.9 (1.9)	2.6 (1.8)	3.4 (2)	3.1 (1.8)	3.5 (2)
Ischemic stroke/stroke unspecified/TIA (%)	35,049 (14)	11,472 (9)	22,859 (20)	1140 (13)	1085 (33)
Ischemic stroke (%)	23,182 (9)	7688 (6)	14,997 (13)	738 (8)	865 (26)
Venous thrombosis (%)	21,066 (9)	9907 (8)	10,689 (9)	775 (9)	488 (15)
Vascular disease (%)	52,831 (22)	13,805 (11)	37,988 (33)	2795 (31)	830 (25)
Any severe bleed (%)	21,045 (9)	9015 (7)	11,411 (10)	1013 (11)	510 (15)
Gastrointestinal bleeding (%)	7435 (3)	3148 (3)	4087 (4)	352 (4)	148 (4)
Hemorrhagic stroke (%)	1335 (< 1)	565 (< 1)	716 (< 1)	39 (< 1)	105 (3)
Heart failure (%)	33,619 (14)	14,095 (12)	18,558 (16)	1718 (19)	558 (17)
Hypertension (%)	113,875 (47)	47,977 (39)	63,059 (54)	5132 (57)	1810 (55)
Other comorbidity					
Cancer (%)	38,589 (16)	16,505 (13)	20,372 (17)	3024 (34)	952 (29)
Anemia (%)	4956 (2)	1930 (2)	2881 (2)	253 (3)	138 (4)
COPD/emphysema (%)	14,138 (6)	5877 (5)	7852 (7)	739 (8)	249 (8)
Diabetes (%)	32,235 (13)	12,285 (10)	19,249 (17)	1405 (16)	532 (16)
Liver disease (%)	2521 (1)	1175 (< 1)	1263 (1)	141 (2)	65 (2)
Renal disease (%)	7958 (3)	3028 (2)	4630 (4)	574 (6)	175 (5)
Dementia (%)	5411 (2)	1993 (2)	3310 (3)	139 (2)	176 (5)
Obesity (%)	2200 (< 1)	892 (< 1)	1246 (1)	130 (1)	42 (1)
Alcohol abuse (%)	1536 (< 1)	723 (< 1)	763 (< 1)	55 (< 1)	57 (2)
Frequent falls (> 2 registrations) (%)	3271 (1)	1572 (1)	1630 (1)	116 (1)	72 (2)

Comorbidities indicate current or relevant previous diagnoses at baseline

Acetylsalicylic acid (ASA) dominated among drugs with a pharmacodynamic effect on bleeding (Table 2, Online Resource, Suppl. Table 4). Furthermore, clopidogrel and non-steroidal anti-inflammatory drugs (NSAIDs) were relatively common in combination with all NOACs. Drugs that interact pharmacokinetically were uncommon in combination with NOACs, generally less than 1 % of patients for each NOAC.

**Table 2:** Potential NOAC drug interactions with drugs with a frequency of ≥ 50 for at least one NOAC at index, in 244,597 patients with newly initiated NOAC treatment during 2008–2017 in Swedish outpatient care. Values are given as *n* (%)

	Apixaban	Rivaroxaban	Dabigatran	Edoxaban
	*n* = 148,108	*n* = 59,276	*n* = 35,617	*n* = 1596
Cardiovascular drugs				
Amiodarone	1368 (0.92)	341 (0.58)	207 (0.58)	38 (2.38)
Diltiazem	528 (0.36)	259 (0.44)	195 (0.55)	9 (0.56)
Dronedarone	1135 (0.77)	168 (0.28)	201 (0.56)	129 (8.08)
Verapamil	962 (0.65)	416 (0.7)	250 (0.7)	16 (1)
Antiepileptics				
Carbamazepine	525 (0.35)	159 (0.27)	113 (0.32)	19 (1.19)
Levetiracetam	651 (0.44)	248 (0.42)	157 (0.44)	8 (0.5)
Phenytoin	118 (0.08)	47 (0.08)	26 (0.07)	4 (0.25)
Valproic acid	365 (0.25)	137 (0.23)	61 (0.17)	3 (0.19)
Antibiotics				
Clarithromycin	53 (0.04)	19 (0.03)	7 (0.02)	1 (0.06)
Rifampicin	56 (0.04)	24 (0.04)	12 (0.03)	5 (0.31)
Antidepressants				
Citalopram	5401 (3.65)	1930 (3.26)	1095 (3.07)	53 (3.32)
Escitalopram	1523 (1.03)	597 (1.01)	282 (0.79)	17 (1.07)
Fluoxetine	438 (0.3)	208 (0.35)	115 (0.32)	3 (0.19)
Paroxetine	473 (0.32)	208 (0.35)	120 (0.34)	7 (0.44)
Sertraline	3498 (2.36)	1392 (2.35)	674 (1.89)	24 (1.5)
Clomipramine	180 (0.12)	70 (0.12)	39 (0.11)	5 (0.31)
Duloxetine	653 (0.44)	282 (0.48)	172 (0.48)	12 (0.75)
Venlafaxine	1212 (0.82)	517 (0.87)	264 (0.74)	19 (1.19)
Antimycotics				
Fluconazole	159 (0.11)	78 (0.13)	21 (0.06)	5 (0.31)
Antineoplastic agents				
Enzalutamide	50 (0.03)	7 (0.01)	10 (0.03)	3 (0.19)
Bicalutamide	1033 (0.7)	352 (0.59)	150 (0.42)	13 (0.81)
Tamoxifen	390 (0.26)	192 (0.32)	87 (0.24)	11 (0.69)
Immunomodulating agents				
Ciclosporin	76 (0.05)	14 (0.02)	4 (0.01)	2 (0.13)
Dexamethasone	205 (0.14)	49 (0.08)	5 (0.01)	3 (0.19)
Prednisone	162 (0.11)	90 (0.15)	19 (0.05)	2 (0.13)
Tacrolimus	174 (0.12)	42 (0.07)	7 (0.02)	5 (0.31)
Analgesic				
Tramadol	1520 (1.03)	1351 (2.28)	2428 (6.82)	11 (0.69)
Antithombotic agents				
Acetylsalicylic acid	39,888 (26.93)	14,168 (23.9)	8541 (23.98)	375 (23.5)
Clopidogrel	7060 (4.77)	2380 (4.02)	1191 (3.34)	69 (4.32)
Dipyridamole	808 (0.55)	211 (0.36)	207 (0.58)	2 (0.13)
Ticagrelor	1202 (0.81)	365 (0.62)	207 (0.58)	17 (1.07)
Dalteparin	3495 (2.36)	1912 (3.23)	535 (1.5)	145 (9.09)
Enoxaparin	571 (0.39)	236 (0.4)	116 (0.33)	16 (1)
Tinzaparin	2488 (1.68)	1026 (1.73)	279 (0.78)	86 (5.39)
NSAIDs				
Celecoxib	1105 (0.75)	154 (0.26)	134 (0.38)	6 (0.38)
Dexibuprofen	120 (0.08)	80 (0.13)	63 (0.18)	1 (0.06)
Diclofenac	2038 (1.38)	1622 (2.74)	1818 (5.1)	7 (0.44)
Etoricoxib	4207 (2.84)	2845 (4.8)	782 (2.2)	6 (0.38)
Ibuprofen	1256 (0.85)	666 (1.12)	559 (1.57)	9 (0.56)
Ketoprofen	781 (0.53)	424 (0.72)	420 (1.18)	4 (0.25)
Meloxicam	59 (0.04)	29 (0.05)	30 (0.08)	0 (0)
Nabumetone	242 (0.16)	87 (0.15)	308 (0.86)	1 (0.06)
Naproxen	3604 (2.43)	1990 (3.36)	1098 (3.08)	19 (1.19)
Tenoxicam	53 (0.04)	30 (0.05)	26 (0.07)	0 (0)

Percentages based on the total number of patients dispensed each NOAC. The full table is available in Online Resource, Suppl. Table 4

**Fig. 2:**
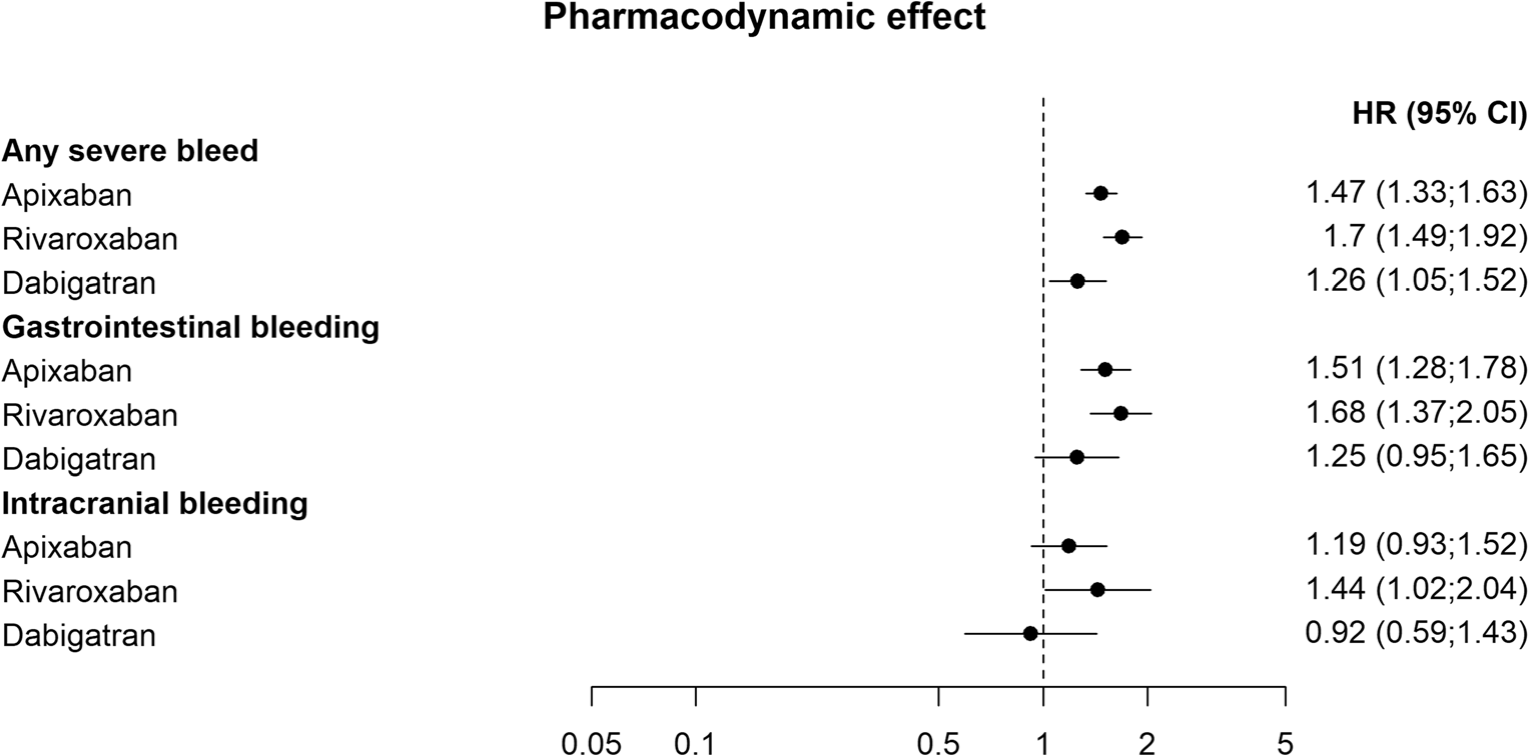
Adjusted hazard ratios of outcomes related to co-treatment with NOACs and interacting drugs with pharmacodynamic effect, compared with NOACs without the interacting drug group

**Fig. 3:**
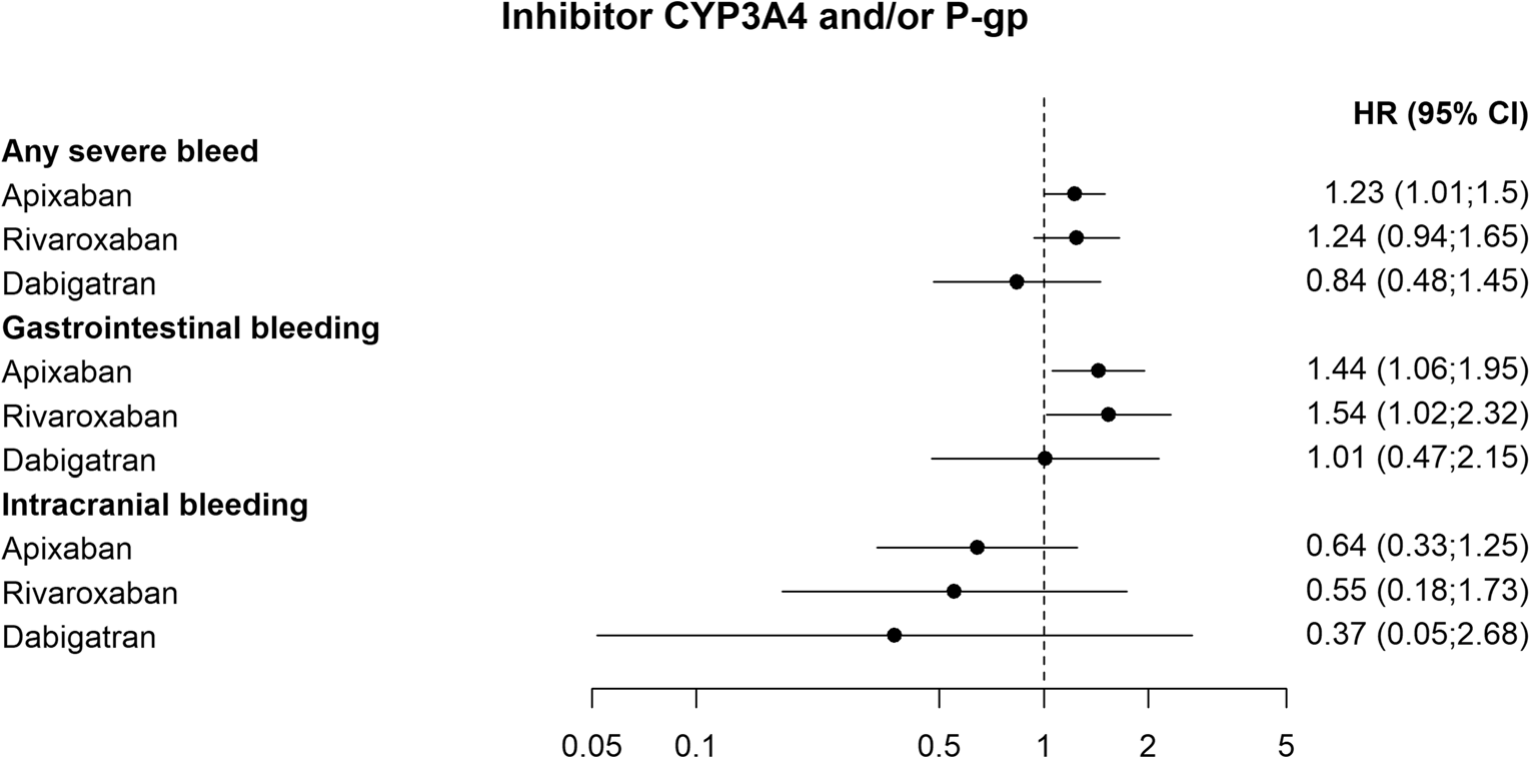
Adjusted hazard ratios of outcomes related to co-treatment with NOACs and CYP3A4 and/or P-gp inhibitors, compared with NOACs without the interacting drug group

In the adjusted Cox-regression analyses, pharmacodynamic and pharmacokinetic interactions with potential effect on the risk of bleeding were associated with an increased risk of any severe bleed. The association with drugs with pharmacodynamic effect appeared to be more pronounced for rivaroxaban (HR 1.7; 1.49–1.92), and apixaban (HR 1.47; 1.33–1.63) than for dabigatran (HR 1.26; 1.05–1.52) (Fig. 2 and Fig. 5). Patients treated with apixaban that were exposed to CYP3A4 and/or P-gp inhibitors also had an increased risk of any severe bleed (HR 1.23; 1.01–1.5), whereas a significant effect for patients treated with rivaroxaban or dabigatran and these interacting drugs could not be established (rivaroxaban, HR 1.24; 0.94–1.65, and dabigatran HR 0.84; 0.48–1.45) (Fig. 3).

**Fig. 4:**
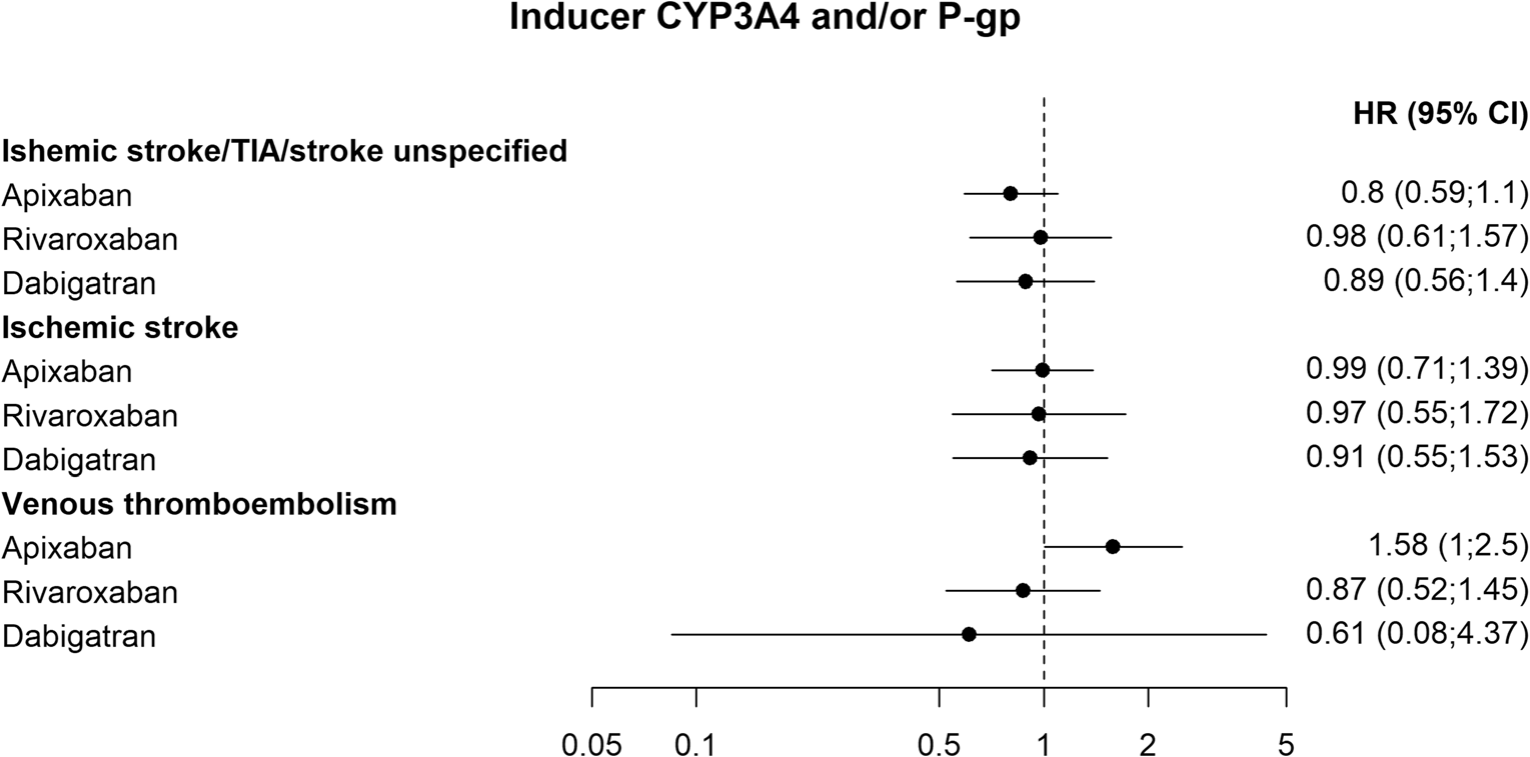
Adjusted hazard ratios of outcomes related to co-treatment with NOACs and CYP3A4 and/or P-gp inducers, compared with NOACs without the interacting drug group

Few patients had inducers of CYP3A4 and/or P-gp in the dataset (Online Resource, Suppl. Table 5). Therefore, estimates of the risk of the composite primary endpoint ischemic stroke/TIA/stroke unspecified associated with this drug group could not be established in the adjusted Cox-regression analyses. Notably however, in this dataset the upper limit of the confidence interval of the endpoint did not exceed 1.57 for any of the NOACs analyzed (Fig. 4).

**Fig. 5:**
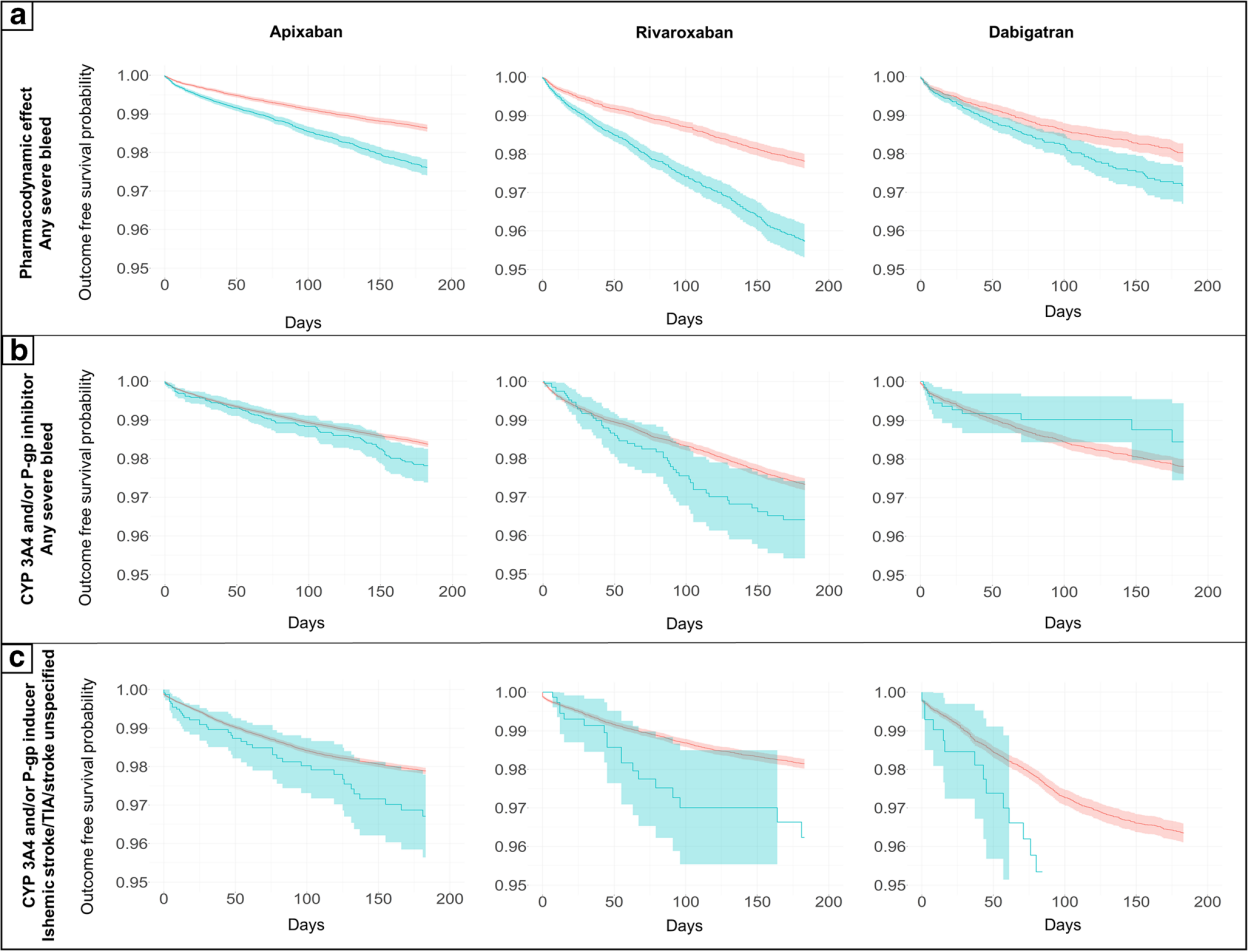
Unadjusted Kaplan-Meier curves of the effect of interactions with pharmacodynamic effect, CYP 3A4 and/or P-gp inhibitor, and CYP 3A4 and/or P-gp inducer, on the primary composite endpoints any severe bleed or ischemic stroke/TIA/stroke unspecified, respectively. Interacting drug group vs. no exposure to interacting drug group for each NOAC. Blue: Exposed to interaction with pharmacodynamic effect, inhibiting- or inducing effect on CYP3A4 and/or P-gp, respectively. Red: Not exposed to interaction with pharmacodynamic effect, inhibiting- or inducing effect on CYP3A4 and/or P-gp, respectively. Note that the y-axis is truncated to 95%. Panel A: Interactions with pharmacodynamic effect. Panel B: Interactions via inhibition of CYP 3A4 and/or P-gp. Panel C: Interactions via induction of CYP 3A4 and/or P-gp

Among the secondary endpoints, the association of drugs that have a pharmacodynamic effect on bleeding and CYP3A4 and/or P-gp-inhibitors were associated with an increased risk of gastrointestinal bleeding for apixaban (HR 1.51; 1.28–1.78, and HR 1.44; 1.06–1.95) and rivaroxaban (HR 1.68; 1.37–2.05, and HR 1.54; 1.02–2.32) (Fig. 2 and Fig. 3). The combined use of apixaban and inducers of CYP3A4 and/or P-gp was associated with an increased risk of venous thromboembolism (HR 1.58; 1.0–2.5) (Fig. 4 and Fig 5).

The proportional hazards assumption and potential interaction between covariables were evaluated. For the analysis of any severe bleed in patients with apixaban and inhibitors of CYP3A4 and/or P-gp, a significant effect of time on the HR was detected. The analysis indicated that the effect may be somewhat larger in the later part of the study period. No statistical interactions were detected between covariables in evaluations preformed for analyses of primary endpoints in the apixaban datasets.

## Discussion

A higher risk of bleeding events was found in patients treated with NOACs and drugs with pharmacodynamic effect on bleeding. The effect of was smaller for dabigatran, and a similar pattern could be seen in secondary endpoints. It should be noted though, that confidence intervals overlap and that we did not formally compare different NOACs in the analysis.

A recently published case-control study of British primary care patients with 393 cases and 1494 controls showed increased risks for major bleeding events in patients treated with any NOAC and drugs with potential pharmacodynamic effect on bleeding [15]. Analyses of individual NOACs in combination with drugs that interact pharmacodynamically indicated higher risk for apixaban and dabigatran but was not statistically significant for rivaroxaban. Individual interacting drugs may have different pharmacodynamic effects on bleeding. The varying results may depend on the distribution of drugs within the group. Furthermore, interactions with a third drug may influence the resulting effect on bleeding. For example patients treated with NOAC and ASA could also be given a proton pump inhibitor (PPI). The concomitant treatment with a PPI lowers dabigatran plasma concentration and may influence the resulting bleeding risk more for dabigatran than for apixaban or rivaroxaban [25].

ASA was very frequent in our dataset, and the effect of this drug can be expected to impact highly on the results of the analyses (Table 2, suppl. 1). Clopidogrel and NSAIDs, in contrast, were not as frequent. In the British cohort mentioned above, the effect was more pronounced for ASA, and not significant for clopidogrel or NSAIDs [15]. Therefore, further comparisons in our material between ASA, P2Y12 receptor blockers, and NSAIDs will be a priority in future work.

Among patients treated with apixaban and inhibitors of CYP3A4 and/or P-gp, there was a higher risk of any severe bleed compared with apixaban alone (Fig. 3). Estimates for rivaroxaban and dabigatran were not significantly different with or without these drug interactions. However, there was a trend indicating higher risk estimates for apixaban and rivaroxaban than for dabigatran, and a similar pattern could be seen in the secondary endpoint, gastrointestinal bleeding, a component of the primary endpoint. Apixaban and rivaroxaban are substrates of CYP3A4 and P-gp, whereas dabigatran is a substrate of P-gp only [13, 26]. However, this potential difference in bleeding risk between different NOACs with regard to metabolic drug interactions should be interpreted with caution and warrants further exploration.

In comparison, no statistically significant effect on major bleeding was seen for inhibitors of CYP3A4 and/or P-gp in the British cohort above [15]. However, in a retrospective study of a Taiwanese cohort of 91,330 NOAC patients with atrial fibrillation, incidence rates of major bleeding were increased for patients with concomitant treatment with NOACs and amiodarone or fluconazole compared with NOAC alone [14]. It should be noted though that the authors also report an association with major bleeding for rifampin and phenytoin, which is unexpected as these drugs are expected to induce NOAC metabolism with lower systemic levels, whereas no increased risk of bleeding was evident for the combined use of NOACs and dronedarone, which is a strong P-gp and CYP3A4 inhibitor. Consequently, the possibility of residual confounding has been discussed [27–29].

The number of patients treated with NOAC and inducers of CYP3A4 and/or P-gp was small in our study, and consequentially the effect of these potential interactions could not be established (Fig. 2). However, the analysis of venous thrombosis among patients treated with apixaban and inducers of CYP3A4 and/or P-gp, which was the largest group of patients analyzed for this endpoint, indicates an increased risk for thromboembolism that needs further exploration.

In a meta-analysis based on data from the ARISTOTLE and ROCKET-AF trials, comparisons with warfarin for apixaban and rivaroxaban are presented stratified on interacting drug groups [12]. The risk of major bleeding was higher for rivaroxaban in combination with at least one combined CYP3A4 and P-gp inhibitor compared with warfarin. For aggregated data of apixaban and rivaroxaban, and analyses of each drug, with drugs affecting CYP3A4 and P-gp metabolism, no other significant differences were found in comparison with warfarin. However, for both stroke and major bleeding, estimates of RRs compared with warfarin were higher for patients with drugs affecting metabolism through CYP3A4 and P-gp than for patients without those drugs in the aggregated data and in separate analyses of apixaban and rivaroxaban.

An important strength of this study was the use of a large nationwide cohort with all outpatient drug use from the introduction of NOAC in 2008, until 2017. In addition, drugs that interact with NOACs were defined based on the Janusmed interactions database and in guidelines from EHRA, sources that are used in the clinical setting in Sweden and Europe [1, 17, 30]. Furthermore, the use of NOACs is increasing and evaluation of risk-benefit aspects of treatment is continuously needed [2, 12, 14, 15].

Patients treated with NOAC without any potentially interacting drugs had lower mean CHA_2_DS_2_-VASc, less cardiovascular morbidity, and lower frequencies of other comorbidities (Table 1). Selected components HAS-BLED and CHA_2_DS_2_-VASc were included as covariables in the analyses, but it is probable that there may be residual confounding that could not be controlled for in this dataset. Data in this study come from large nationwide registers, which is a strength from a power perspective. However, the limited information in the registers on the specific health status of each patient is a limitation. In addition, OTC drugs, e.g., ASA and NSAIDs, are not included in the Prescribed Drug Register, and the use of these drugs may differ in different patient groups. Furthermore, we did not have access to specific dosing information. Therefore, analyses could not be controlled for dose adjustment in NOACs or the potentially interacting drugs. Dose adjustment is a way to handle drug-drug interactions clinically and may lead to an exposure and risk profile that is similar to patients not treated with the interacting drug, which in turn may lead to reduced effect estimates in our study [1].

Analyses of the effect of CYP3A4 and/or P-gp inducers on the risk of thromboembolic events failed to show any difference in risk compared with patients without those drugs. Also, in some cases, HRs were lower for the patients exposed to interacting drugs, but with wide confidence intervals allowing for the possibility that an effect of the interaction may exist but could not be estimated. Due to the relatively small number of patients treated with these drugs in the cohort, this was not an unexpected result of the analyses (Online Resource, Suppl. Table 5).

In this study, we chose to analyze the risk of bleeding or thromboembolism depending on the expected interaction effect. However, an alternative approach could have been to analyze the net clinical risk and to use a composite endpoint with both bleeding and thromboembolism. Such a design would have the benefit of quantifying the general risk but would not have allowed an evaluation of the clinical effect related to the expected mechanism of different drug interactions.

Censoring was performed based on cessation of drug treatment or death. We did not include competing risks in the analyses. The rationale for this was based on the consideration that drug treatment with NOAC may be discontinued or not depending on the clinical situation, and therefore an accurate definition of relevant competing events could not easily be identified.

We performed multiple analyses of different combinations of drugs in this observational study, and therefore multiple testing may be considered an issue. Whether adjustment for multiple testing should be performed in observational studies or not have been discussed, and different opinions exist on the matter [31, 32]. We chose not to adjust for multiple testing and the results should be considered exploratory.

## Conclusion

The results of this nationwide study indicate a higher risk of any severe bleed for patients exposed to drugs that interact pharmacodynamically with NOACs. Furthermore, inhibitors of CYP3A4 and/or P-gp increased the risk of bleeding, whereas results for inducers of CYP3A4 and or P-gp were limited due to the small number of exposed patients. In conclusion, the increased risk with co-prescription of NOACs and interacting drugs is important to consider in clinical practice. Furthermore, to evaluate risks in large groups of patients, additional evaluations in observational studies, in particular focusing on less common combinations with potential clinical relevance, are needed as NOAC use increase with time.

## Electronic supplementary material

ESM 1 (PDF 403 kb)

## Data Availability

The data that support the findings of this study are available from the Swedish National Board of Health and Welfare. Restrictions apply to the availability of these data, which were used under license for this study. Data are available from the authors upon reasonable request and with the permission of the National Board of Health and Welfare.
