# Supplementary material for: Bleeding and thromboembolism due to drug-drug interactions with non-vitamin K antagonist oral anticoagulants—a Swedish, register-based cohort study in atrial fibrillation outpatients
Source: Eur J Clin Pharmacol. 2020 Oct 7;77(3):409–19. doi: 10.1007/s00228-020-03015-7 (PMC7867544; doi:10.1007/s00228-020-03015-7)
Supplement: Supplementary file 1 — (PDF 403 kb) [file 228_2020_3015_MOESM1_ESM.pdf]

Supplementary material

Supplementary table 1. Definitions of medical conditions by ICD-10 codes and procedure codes, for descriptive purposes, covariates and outcomes.

Variable	ICD-diagnoses and procedure codes	Category[b]
Atrial fibrillation	I48	1
Mechanical valve	Z952, FCA60, FDC10, FGE00, FGE96, FJF00, FJF96, FKD00, FKD96, FMD00, FMD96	1
Mitral stenosis	I050, I342, I052, Q232	1
Heart failure	I110, I130, I132, I50	1
Hypertension	I10, I11, I12, I13, I15, O10	1
Diabetes	E10, E11, E12, E13, E14, G590, G632, H280, H360, M142, N083	1
TIA/RIND	G45, Z866A[a], Z866B[a]	1
Stroke unspecified	I64, I694[a], Z867C[a]	1
Ischemic stroke	I63, I693[a]	1
Arterial embolism and thrombosis	I74	1
Vascular disease	I20, I21, I22, I23, I24, I25, I67, I70, I71, I72, I73, M311, K550, K551, K559	1
Cancer	C	2
Dementia	F00, F01, F02, F03, F051, F107A, G30, G318A	1
Alcohol abuse	E244, F10, G312, G621, G721, I426, K292, K70, K852, K860, O354, T510, T519, Y90, Y91, Z502, Z714, Z721	3
Anemia	D46, D50, D51, D52, D53, D55, D56, D57, D58, D59, D60, D61, D62, D63, D64, O990	3
Venous thrombosis	I26, I80, I81, I82, K645, O223, O871, Z867A[a], Z867B[a]	1
DVT/PE	I26, I80, O223, O871	4
Hip or knee replacement	NFB, NFC, NGB, NGC	4
Renal disease	N18, N19, Y841, Z992, N17	1, N17: 3
Liver disease	B150, B160, B162, B180E, B180G, B181E, B181G, B182E, B182G, B188E, B188G, B189E, B189G, B190, K70, K71, K72, K73, K74, K75, K76, K77	1
Obesity	E65, E66	3
COPD/emphysema	J43, J44, J982, J983	1
Gastrointestinal bleeding	I850, I983, K226, K250, K251, K252, K254, K255, K256, K260, K261, K262, K264, K265, K266, K270, K271, K272, K274, K275, K276, K280, K281, K282, K284, K285, K286, K290, K625, K920, K921, K922	2
Hemorrhagic stroke	I61	2
Other intracranial bleeding	I60, I62, S064, S065, S066	2
Other severe bleeding	D500, D62, D683, D698, D699, H313, H356, H431, I312, J942, K661, N501A, N93, N950, O20, R041, R042, R048, R049, R58, T810	2
Fall	R296, W00, W01, W02, W03, W04, W05, W06, W07, W08, W09, W10, W11, W12, W13, W14, W15, W17, W18, W19	3
Ischemic stroke/TIA/arterial embolus/stroke unspecified	I63, I693[a], G45, Z866A[a], Z866B[a], I74, I64, I694[a], Z867C[a]	1
Ischemic stroke/stroke unspecified/TIA	I63, I693[a], G45, Z866A[a], Z866B[a], I64, I694[a], Z867C[a]	1

Any severe bleed	I850, I983, K226, K250, K251, K252, K254, K255, K256, K260, K261, K262, K264, K265, K266, K270, K271, K272, K274, K275, K276, K280, K281, K282, K284, K285, K286, K290, K625, K920, K921, K922, I61, I60, I62, S064, S065, S066, D500, D62, D683, D698, D699, H313, H356, H431, I312, J942, K661, N501A, N93, N950, O20, R041, R042, R048, R049, R58, T810	2
Intracranial bleeding	I61, I60, I62, S064, S065, S066	2

[a] Diagnoses that indicate historical event, used for covariates and descriptive variables, but not for outcomes.

[b] Categories 1-3 indicate the time period before index date for which an occurrence of the diagnosis was considered relevant for covariates and descriptive variables. 1 = 10 years, 2 = 5 years, 3 = 6 months. Category 4 was considered within 60 days before index date as exclusion criteria.

Supplementary table 2. Definitions of drug treatment by ATC-codes.

Subtance	ATC-codes
abiraterone	L02BX03
aceclofenac	M01AB16
acemetacin	M01AB11
acenocoumarol	B01AA07
acetylsalicylic acid	B01AC06, B01AC56, C07FX02, C07FX03, C07FX04, C10BX01, C10BX02, C10BX04, C10BX05, C10BX06, C10BX08, C10BX12, M01BA03, N02AJ02, N02AJ07, N02AJ18, N02BA01, N02BA51, N02BA71
amiodarone	C01BD01
amprenavir	J05AE05
apalutamide	L02BB05
apixaban	B01AF02
argatroban	B01AE03
atazanavir	J05AE08, J05AR15, J05AR23
benzydamine	M01AX07
bicalutamide	L02AE51, L02BB03
bivalirudin	B01AE06
boceprevir	J05AE12, J05AP03
caplacizumab	B01AX07
carbamazepine	N03AF01
cariprazine	N05AX15
celecoxib	L01XX33, M01AH01
ciclosporin	L04AA01, L04AD01
cilostazol	B01AC23
citalopram	N06AB04
clarithromycin	A02BD04, A02BD05, A02BD06, A02BD07, A02BD09, A02BD11, J01FA09
clomipramine	N06AA04
clopidogrel	B01AC04
cobicistat	J05AR09, J05AR14, J05AR15, J05AR18, J05AR22, V03AX03
crizotinib	L01XE16
dabigatran	B01AE07
dalteparin	B01AB04
danaparoid	B01AB09
darunavir	J05AE10, J05AR14, J05AR22
defibrotide	B01AX01
desirudin	B01AE01
dexamethasone	H02AB02
dexibuprofen	M01AE14
dexketoprofen	M01AE17, N02AJ14
diclofenac	M01AB05, M01AB55
diflunisal	N02BA11
diltiazem	C08DB01
dipyridamole	B01AC07
doxorubicin	L01DB01

dronedarone	C01BD07
duloxetine	N06AX21
edoxaban	B01AF03
efavirenz	J05AG03, J05AR06, J05AR11
enoxaparin	B01AB05
enzalutamide	L02BB04
epoprostenol	B01AC09
erythromycin	J01FA01, S01AA17
escitalopram	N06AB10
etodolac	M01AB08
etoricoxib	M01AH05
fluconazole	J01RA07, J02AC01
fluoxetine	N06AB03, N06CA03
flurbiprofen	M01AE09
fluvoxamine	N06AB08
fondaparinux	B01AX05
fosamprenavir	J05AE07
glecaprevir	J05AP57
heparin	B01AB01, B01AB51
ibrutinib	L01XE27
ibuprofen	C01EB16, M01AE01, M01AE51, N02AJ08, N02AJ19
idelalisib	L01XX47
iloprost	B01AC11
imatinib	L01XE01, L01XX28
indinavir	J05AE02
indobufen	B01AC10
indometacin	C01EB03, M01AB01, M01AB51
inotuzumab ozogamicin	L01XC26
ipilimumab	L01XC11
itraconazole	J02AC02
kebuzone	M01AA06
ketoconazole	G01AF11, J02AB02
ketoprofen	M01AE03, M01AE53
ketorolac	M01AB15
lapatinib	L01XE07
lepirudin	B01AE02
letermovir	J05AX18
levetiracetam	N03AX14
lopinavir	J05AE06, J05AR10
lornoxicam	M01AC05
lumacaftor	R07AX30
lumiracoxib	M01AH06
meclofenamic acid	M01AG04
mefenamic acid	M01AG01
meloxicam	M01AC06, M01AC56

miconazole	A07AC01, G01AF04, J02AB01, S02AA13
milnacipran	N06AX17
mitotane	L01XX23
nabumetone	M01AX01
nadroparin	B01AB06
naproxen	M01AE02, M01AE52, M01AE56
nelfinavir	J05AE04
neratinib	L01XE45
niflumic acid	M01AX02
nilotinib	L01XE08
nimesulide	M01AX17
oxcarbazepine	N03AF02
paclitaxel	L01CD01, L01CD03
parecoxib	M01AH04
paroxetine	N06AB05
pentosan polysulfate	G04BX15
phenobarbital	N03AA02
phenprocoumon	B01AA04
phenylbutazone	M01AA01, M01BA01
phenytoin	N03AB02, N03AB52
pibrentasvir	J05AP57
piroxicam	M01AC01, S01BC06
posaconazole	J02AC04
prasugrel	B01AC22
prednisone	H02AB07
primidone	N03AA03
proglumetacin	M01AB14
propafenone	C01BC03
quinidine	C01BA01, C01BA51, C01BA71
ramucirumab	L01XC21
reviparin	B01AB08
rifampicin	J04AB02, J04AM02, J04AM05, J04AM06, J04AM07
rifamycin	J04AB03, S01AA16, S02AA12
ritonavir	J05AE03, J05AE06, J05AP52, J05AP53, J05AR10, J05AR23, J05AX66, J05AX67
rivaroxaban	B01AF01, B01AX06
rofecoxib	M01AH02
rolapitant	A04AD14
saquinavir	J05AE01
sarilumab	L04AC14
sertraline	N06AB06
siltuximab	L04AC11
sulindac	M01AB02
sunitinib	L01XE04
tacrolimus	L04AA05, L04AD02
tamoxifen	L02BA01

telaprevir	J05AE11, J05AP02
telithromycin	J01FA15
tenoxicam	M01AC02
tiaprofenic acid	M01AE11
ticagrelor	B01AC24
ticlopidine	B01AC05
tinzaparin	B01AB10
tipranavir	J05AE09
tocilizumab	L04AC07
tolfenamic acid	M01AG02
topiramate	N03AX11
tramadol	N02AJ13, N02AJ14, N02AJ15, N02AX02, N02AX52
treprostinil	B01AC21
troleandomycin	J01FA08
valdecoxib	M01AH03
valproic acid	N03AG01
vandetanib	L01XE12
warfarin	B01AA03
velpatasvir	J05AP55, J05AP56, J05AX69
venetoclax	L01XX52
venlafaxine	N06AX16
verapamil	C08DA01, C08DA51, C09BB10
vinblastine	L01CA01
vorapaxar	B01AC26
voriconazole	J02AC03
voxilaprevir	J05AP56

Supplementary table 3. Substances included in interacting drug groups per NOAC. Included substances based on documented clinically relevant interactions in the Janusmed Interaction Database or by EHRA [ref].

Substance	Apixaban	Dabigatran	Edoxaban	Rivaroxaban
abiraterone	CYP3A4/P-gp inhibitor	CYP3A4/P-gp inhibitor	CYP3A4/P-gp inhibitor	CYP3A4/P-gp inhibitor
aceclofenac	Pharmacodynamic effect	Pharmacodynamic effect	Pharmacodynamic effect	Pharmacodynamic effect
acemetacin	Pharmacodynamic effect	Pharmacodynamic effect	Pharmacodynamic effect	Pharmacodynamic effect
acenocoumarol	Pharmacodynamic effect	Pharmacodynamic effect	Pharmacodynamic effect	Pharmacodynamic effect
acetylsalicylic acid	Pharmacodynamic effect	Pharmacodynamic effect	Pharmacodynamic effect	Pharmacodynamic effect
amiodarone	CYP3A4/P-gp inhibitor	CYP3A4/P-gp inhibitor	CYP3A4/P-gp inhibitor	CYP3A4/P-gp inhibitor
amprenavir	CYP3A4/P-gp inhibitor	CYP3A4/P-gp inhibitor	CYP3A4/P-gp inhibitor	CYP3A4/P-gp inhibitor
apalutamide	NA	CYP3A4/P-gp inducer	NA	NA
apixaban	NA	Pharmacodynamic effect	Pharmacodynamic effect	Pharmacodynamic effect
argatroban	Pharmacodynamic effect	Pharmacodynamic effect	Pharmacodynamic effect	Pharmacodynamic effect
atazanavir	CYP3A4/P-gp inhibitor	CYP3A4/P-gp inhibitor	CYP3A4/P-gp inhibitor	CYP3A4/P-gp inhibitor
benzydamine	Pharmacodynamic effect	Pharmacodynamic effect	Pharmacodynamic effect	Pharmacodynamic effect
bicalutamide	CYP3A4/P-gp inhibitor	NA	NA	CYP3A4/P-gp inhibitor
bivalirudin	Pharmacodynamic effect	Pharmacodynamic effect	Pharmacodynamic effect	Pharmacodynamic effect
boceprevir	CYP3A4/P-gp inhibitor	CYP3A4/P-gp inhibitor	CYP3A4/P-gp inhibitor	CYP3A4/P-gp inhibitor
caplacizumab	Pharmacodynamic effect	Pharmacodynamic effect	Pharmacodynamic effect	Pharmacodynamic effect
carbamazepine	CYP3A4/P-gp inducer	CYP3A4/P-gp inducer	NA	CYP3A4/P-gp inducer
cariprazine	NA	CYP3A4/P-gp inhibitor	CYP3A4/P-gp inhibitor	NA
celecoxib	Pharmacodynamic effect	Pharmacodynamic effect	Pharmacodynamic effect	Pharmacodynamic effect
ciclosporin	CYP3A4/P-gp inhibitor	CYP3A4/P-gp inhibitor	CYP3A4/P-gp inhibitor	CYP3A4/P-gp inhibitor
cilostazol	Pharmacodynamic effect	Pharmacodynamic effect	Pharmacodynamic effect	Pharmacodynamic effect
citalopram	Pharmacodynamic effect	Pharmacodynamic effect	Pharmacodynamic effect	Pharmacodynamic effect
clarithromycin	CYP3A4/P-gp inhibitor	CYP3A4/P-gp inhibitor	CYP3A4/P-gp inhibitor	CYP3A4/P-gp inhibitor
clomipramine	Pharmacodynamic effect	Pharmacodynamic effect	Pharmacodynamic effect	Pharmacodynamic effect
clopidogrel	Pharmacodynamic effect	Pharmacodynamic effect	Pharmacodynamic effect	Pharmacodynamic effect
cobicistat	CYP3A4/P-gp inhibitor	CYP3A4/P-gp inhibitor	CYP3A4/P-gp inhibitor	CYP3A4/P-gp inhibitor
crizotinib	CYP3A4/P-gp inhibitor	CYP3A4/P-gp inhibitor	CYP3A4/P-gp inhibitor	CYP3A4/P-gp inhibitor
dabigatran	Pharmacodynamic effect	NA	Pharmacodynamic effect	Pharmacodynamic effect
dalteparin	Pharmacodynamic effect	Pharmacodynamic effect	Pharmacodynamic effect	Pharmacodynamic effect
danaparoid	Pharmacodynamic effect	Pharmacodynamic effect	Pharmacodynamic effect	Pharmacodynamic effect
darunavir	CYP3A4/P-gp inhibitor	CYP3A4/P-gp inhibitor	CYP3A4/P-gp inhibitor	CYP3A4/P-gp inhibitor
defibrotide	Pharmacodynamic effect	Pharmacodynamic effect	Pharmacodynamic effect	Pharmacodynamic effect
desirudin	Pharmacodynamic effect	Pharmacodynamic effect	Pharmacodynamic effect	Pharmacodynamic effect
dexamethasone	CYP3A4/P-gp inducer	CYP3A4/P-gp inducer	CYP3A4/P-gp inducer	CYP3A4/P-gp inducer
dexibuprofen	Pharmacodynamic effect	Pharmacodynamic effect	Pharmacodynamic effect	Pharmacodynamic effect
dexketoprofen	Pharmacodynamic effect	Pharmacodynamic effect	Pharmacodynamic effect	Pharmacodynamic effect
diclofenac	Pharmacodynamic effect	Pharmacodynamic effect	Pharmacodynamic effect	Pharmacodynamic effect
diflunisal	Pharmacodynamic effect	Pharmacodynamic effect	Pharmacodynamic effect	Pharmacodynamic effect
diltiazem	CYP3A4/P-gp inhibitor	CYP3A4/P-gp inhibitor	CYP3A4/P-gp inhibitor	CYP3A4/P-gp inhibitor
dipyridamole	Pharmacodynamic effect	Pharmacodynamic effect	Pharmacodynamic effect	Pharmacodynamic effect

doxorubicin	CYP3A4/P-gp inducer	CYP3A4/P-gp inducer	CYP3A4/P-gp inducer	CYP3A4/P-gp inducer
dronedarone	CYP3A4/P-gp inhibitor	CYP3A4/P-gp inhibitor	CYP3A4/P-gp inhibitor	CYP3A4/P-gp inhibitor
duloxetine	Pharmacodynamic effect	Pharmacodynamic effect	Pharmacodynamic effect	Pharmacodynamic effect
edoxaban	Pharmacodynamic effect	Pharmacodynamic effect	NA	Pharmacodynamic effect
efavirenz	CYP3A4/P-gp inducer	NA	NA	CYP3A4/P-gp inducer
enoxaparin	Pharmacodynamic effect	Pharmacodynamic effect	Pharmacodynamic effect	Pharmacodynamic effect
enzalutamide	CYP3A4/P-gp inducer	CYP3A4/P-gp inducer	CYP3A4/P-gp inducer	CYP3A4/P-gp inducer
epoprostenol	Pharmacodynamic effect	Pharmacodynamic effect	Pharmacodynamic effect	Pharmacodynamic effect
erythromycin	CYP3A4/P-gp inhibitor	CYP3A4/P-gp inhibitor	CYP3A4/P-gp inhibitor	CYP3A4/P-gp inhibitor
escitalopram	Pharmacodynamic effect	Pharmacodynamic effect	Pharmacodynamic effect	Pharmacodynamic effect
etodolac	Pharmacodynamic effect	Pharmacodynamic effect	Pharmacodynamic effect	Pharmacodynamic effect
etoricoxib	Pharmacodynamic effect	Pharmacodynamic effect	Pharmacodynamic effect	Pharmacodynamic effect
fluconazole	CYP3A4/P-gp inhibitor	NA	NA	CYP3A4/P-gp inhibitor
fluoxetine	Pharmacodynamic effect	Pharmacodynamic effect	Pharmacodynamic effect	Pharmacodynamic effect
flurbiprofen	Pharmacodynamic effect	Pharmacodynamic effect	Pharmacodynamic effect	Pharmacodynamic effect
fluvoxamine	Pharmacodynamic effect	Pharmacodynamic effect	Pharmacodynamic effect	Pharmacodynamic effect
fondaparinux	Pharmacodynamic effect	Pharmacodynamic effect	Pharmacodynamic effect	Pharmacodynamic effect
fosamprenavir	CYP3A4/P-gp inhibitor	CYP3A4/P-gp inhibitor	CYP3A4/P-gp inhibitor	CYP3A4/P-gp inhibitor
glecaprevir	NA	CYP3A4/P-gp inhibitor	NA	NA
heparin	Pharmacodynamic effect	Pharmacodynamic effect	Pharmacodynamic effect	Pharmacodynamic effect
ibrutinib	Pharmacodynamic effect	Pharmacodynamic effect	Pharmacodynamic effect	Pharmacodynamic effect
ibuprofen	Pharmacodynamic effect	Pharmacodynamic effect	Pharmacodynamic effect	Pharmacodynamic effect
idelalisib	CYP3A4/P-gp inhibitor	NA	NA	CYP3A4/P-gp inhibitor
iloprost	Pharmacodynamic effect	Pharmacodynamic effect	Pharmacodynamic effect	Pharmacodynamic effect
imatinib	CYP3A4/P-gp inhibitor	CYP3A4/P-gp inhibitor	CYP3A4/P-gp inhibitor	CYP3A4/P-gp inhibitor
indinavir	CYP3A4/P-gp inhibitor	CYP3A4/P-gp inhibitor	CYP3A4/P-gp inhibitor	CYP3A4/P-gp inhibitor
indobufen	Pharmacodynamic effect	Pharmacodynamic effect	Pharmacodynamic effect	Pharmacodynamic effect
indometacin	Pharmacodynamic effect	Pharmacodynamic effect	Pharmacodynamic effect	Pharmacodynamic effect
inotuzumab ozogamicin	Pharmacodynamic effect	Pharmacodynamic effect	Pharmacodynamic effect	Pharmacodynamic effect
ipilimumab	Pharmacodynamic effect	Pharmacodynamic effect	Pharmacodynamic effect	Pharmacodynamic effect
itraconazole	CYP3A4/P-gp inhibitor	CYP3A4/P-gp inhibitor	CYP3A4/P-gp inhibitor	CYP3A4/P-gp inhibitor
kebuzone	Pharmacodynamic effect	Pharmacodynamic effect	Pharmacodynamic effect	Pharmacodynamic effect
ketoconazole	CYP3A4/P-gp inhibitor	CYP3A4/P-gp inhibitor	CYP3A4/P-gp inhibitor	CYP3A4/P-gp inhibitor
ketoprofen	Pharmacodynamic effect	Pharmacodynamic effect	Pharmacodynamic effect	Pharmacodynamic effect
ketorolac	Pharmacodynamic effect	Pharmacodynamic effect	Pharmacodynamic effect	Pharmacodynamic effect
lapatinib	CYP3A4/P-gp inhibitor	CYP3A4/P-gp inhibitor	CYP3A4/P-gp inhibitor	CYP3A4/P-gp inhibitor
lepirudin	Pharmacodynamic effect	Pharmacodynamic effect	Pharmacodynamic effect	Pharmacodynamic effect
letermovir	NA	CYP3A4/P-gp inducer	NA	NA
levetiracetam	CYP3A4/P-gp inducer	CYP3A4/P-gp inducer	CYP3A4/P-gp inducer	CYP3A4/P-gp inducer
lopinavir	CYP3A4/P-gp inhibitor	CYP3A4/P-gp inhibitor	CYP3A4/P-gp inhibitor	CYP3A4/P-gp inhibitor
lornoxicam	Pharmacodynamic effect	Pharmacodynamic effect	Pharmacodynamic effect	Pharmacodynamic effect
lumacaftor	CYP3A4/P-gp inducer	NA	NA	CYP3A4/P-gp inducer
lumiracoxib	Pharmacodynamic effect	Pharmacodynamic effect	Pharmacodynamic effect	Pharmacodynamic effect
meclofenamic acid	Pharmacodynamic effect	Pharmacodynamic effect	Pharmacodynamic effect	Pharmacodynamic effect
mefenamic acid	Pharmacodynamic effect	Pharmacodynamic effect	Pharmacodynamic effect	Pharmacodynamic effect

meloxicam	Pharmacodynamic effect	Pharmacodynamic effect	Pharmacodynamic effect	Pharmacodynamic effect
miconazole	CYP3A4/P-gp inhibitor	NA	NA	CYP3A4/P-gp inhibitor
milnacipran	Pharmacodynamic effect	Pharmacodynamic effect	Pharmacodynamic effect	Pharmacodynamic effect
mitotane	CYP3A4/P-gp inducer	NA	NA	CYP3A4/P-gp inducer
nabumetone	Pharmacodynamic effect	Pharmacodynamic effect	Pharmacodynamic effect	Pharmacodynamic effect
nadroparin	Pharmacodynamic effect	Pharmacodynamic effect	Pharmacodynamic effect	Pharmacodynamic effect
naproxen	Pharmacodynamic effect	Pharmacodynamic effect	Pharmacodynamic effect	Pharmacodynamic effect
nelfinavir	CYP3A4/P-gp inhibitor	CYP3A4/P-gp inhibitor	CYP3A4/P-gp inhibitor	CYP3A4/P-gp inhibitor
neratinib	NA	CYP3A4/P-gp inhibitor	CYP3A4/P-gp inhibitor	NA
niflumic acid	Pharmacodynamic effect	Pharmacodynamic effect	Pharmacodynamic effect	Pharmacodynamic effect
nilotinib	CYP3A4/P-gp inhibitor	CYP3A4/P-gp inhibitor	CYP3A4/P-gp inhibitor	CYP3A4/P-gp inhibitor
nimesulide	Pharmacodynamic effect	Pharmacodynamic effect	Pharmacodynamic effect	Pharmacodynamic effect
oxcarbazepine	CYP3A4/P-gp inducer	NA	NA	CYP3A4/P-gp inducer
paclitaxel	CYP3A4/P-gp inducer	NA	NA	CYP3A4/P-gp inducer
parecoxib	Pharmacodynamic effect	Pharmacodynamic effect	Pharmacodynamic effect	Pharmacodynamic effect
paroxetine	Pharmacodynamic effect	Pharmacodynamic effect	Pharmacodynamic effect	Pharmacodynamic effect
pentosan polysulfate	Pharmacodynamic effect	Pharmacodynamic effect	Pharmacodynamic effect	Pharmacodynamic effect
phenobarbital	CYP3A4/P-gp inducer	CYP3A4/P-gp inducer	CYP3A4/P-gp inducer	CYP3A4/P-gp inducer
phenprocoumon	Pharmacodynamic effect	Pharmacodynamic effect	Pharmacodynamic effect	Pharmacodynamic effect
phenylbutazone	Pharmacodynamic effect	Pharmacodynamic effect	Pharmacodynamic effect	Pharmacodynamic effect
phenytoin	CYP3A4/P-gp inducer	CYP3A4/P-gp inducer	CYP3A4/P-gp inducer	CYP3A4/P-gp inducer
pibrentasvir	NA	CYP3A4/P-gp inhibitor	NA	NA
piroxicam	Pharmacodynamic effect	Pharmacodynamic effect	Pharmacodynamic effect	Pharmacodynamic effect
posaconazole	CYP3A4/P-gp inhibitor	CYP3A4/P-gp inhibitor	CYP3A4/P-gp inhibitor	CYP3A4/P-gp inhibitor
prasugrel	Pharmacodynamic effect	Pharmacodynamic effect	Pharmacodynamic effect	Pharmacodynamic effect
prednisone	CYP3A4/P-gp inducer	NA	NA	CYP3A4/P-gp inducer
primidone	CYP3A4/P-gp inducer	NA	NA	CYP3A4/P-gp inducer
proglumetacin	Pharmacodynamic effect	Pharmacodynamic effect	Pharmacodynamic effect	Pharmacodynamic effect
propafenone	CYP3A4/P-gp inhibitor	CYP3A4/P-gp inhibitor	CYP3A4/P-gp inhibitor	CYP3A4/P-gp inhibitor
quinidine	CYP3A4/P-gp inhibitor	CYP3A4/P-gp inhibitor	CYP3A4/P-gp inhibitor	CYP3A4/P-gp inhibitor
ramucirumab	Pharmacodynamic effect	Pharmacodynamic effect	Pharmacodynamic effect	Pharmacodynamic effect
reviparin	Pharmacodynamic effect	Pharmacodynamic effect	Pharmacodynamic effect	Pharmacodynamic effect
rifampicin	CYP3A4/P-gp inducer	CYP3A4/P-gp inducer	NA	CYP3A4/P-gp inducer
rifamycin	CYP3A4/P-gp inducer	CYP3A4/P-gp inducer	NA	CYP3A4/P-gp inducer
ritonavir	CYP3A4/P-gp inhibitor	CYP3A4/P-gp inhibitor	CYP3A4/P-gp inhibitor	CYP3A4/P-gp inhibitor
rivaroxaban	Pharmacodynamic effect	Pharmacodynamic effect	Pharmacodynamic effect	NA
rofecoxib	Pharmacodynamic effect	Pharmacodynamic effect	Pharmacodynamic effect	Pharmacodynamic effect
rolapitant	NA	CYP3A4/P-gp inhibitor	CYP3A4/P-gp inhibitor	NA
saquinavir	CYP3A4/P-gp inhibitor	CYP3A4/P-gp inhibitor	CYP3A4/P-gp inhibitor	CYP3A4/P-gp inhibitor
sarilumab	CYP3A4/P-gp inducer	NA	NA	CYP3A4/P-gp inducer
sertraline	Pharmacodynamic effect	Pharmacodynamic effect	Pharmacodynamic effect	Pharmacodynamic effect
siltuximab	CYP3A4/P-gp inducer	NA	CYP3A4/P-gp inducer	CYP3A4/P-gp inducer
sulindac	Pharmacodynamic effect	Pharmacodynamic effect	Pharmacodynamic effect	Pharmacodynamic effect
sunitinib	CYP3A4/P-gp inducer	CYP3A4/P-gp inducer	CYP3A4/P-gp inducer	CYP3A4/P-gp inducer
tacrolimus	CYP3A4/P-gp inhibitor	CYP3A4/P-gp inhibitor	CYP3A4/P-gp inhibitor	CYP3A4/P-gp inhibitor

tamoxifen	CYP3A4/P-gp inhibitor	CYP3A4/P-gp inhibitor	CYP3A4/P-gp inhibitor	CYP3A4/P-gp inhibitor
telaprevir	CYP3A4/P-gp inhibitor	CYP3A4/P-gp inhibitor	CYP3A4/P-gp inhibitor	CYP3A4/P-gp inhibitor
telithromycin	CYP3A4/P-gp inhibitor	CYP3A4/P-gp inhibitor	CYP3A4/P-gp inhibitor	CYP3A4/P-gp inhibitor
tenoxicam	Pharmacodynamic effect	Pharmacodynamic effect	Pharmacodynamic effect	Pharmacodynamic effect
tiaprofenic acid	Pharmacodynamic effect	Pharmacodynamic effect	Pharmacodynamic effect	Pharmacodynamic effect
ticagrelor	Pharmacodynamic effect	Pharmacodynamic effect	Pharmacodynamic effect	Pharmacodynamic effect
ticlopidine	Pharmacodynamic effect	Pharmacodynamic effect	Pharmacodynamic effect	Pharmacodynamic effect
tinzaparin	Pharmacodynamic effect	Pharmacodynamic effect	Pharmacodynamic effect	Pharmacodynamic effect
tipranavir	CYP3A4/P-gp inhibitor	CYP3A4/P-gp inhibitor	CYP3A4/P-gp inhibitor	CYP3A4/P-gp inhibitor
tocilizumab	CYP3A4/P-gp inducer	NA	NA	CYP3A4/P-gp inducer
tolfenamic acid	Pharmacodynamic effect	Pharmacodynamic effect	Pharmacodynamic effect	Pharmacodynamic effect
topiramate	CYP3A4/P-gp inducer	NA	NA	CYP3A4/P-gp inducer
tramadol	Pharmacodynamic effect	Pharmacodynamic effect	Pharmacodynamic effect	Pharmacodynamic effect
treprostinil	Pharmacodynamic effect	Pharmacodynamic effect	Pharmacodynamic effect	Pharmacodynamic effect
troleandomycin	CYP3A4/P-gp inhibitor	NA	NA	CYP3A4/P-gp inhibitor
valdecoxib	Pharmacodynamic effect	Pharmacodynamic effect	Pharmacodynamic effect	Pharmacodynamic effect
valproic acid	CYP3A4/P-gp inducer	CYP3A4/P-gp inducer	CYP3A4/P-gp inducer	CYP3A4/P-gp inducer
vandetanib	CYP3A4/P-gp inducer	CYP3A4/P-gp inducer	CYP3A4/P-gp inducer	CYP3A4/P-gp inducer
warfarin	Pharmacodynamic effect	Pharmacodynamic effect	Pharmacodynamic effect	Pharmacodynamic effect
velpatasvir	NA	CYP3A4/P-gp inhibitor	NA	NA
venetoclax	NA	CYP3A4/P-gp inhibitor	NA	NA
venlafaxine	Pharmacodynamic effect	Pharmacodynamic effect	Pharmacodynamic effect	Pharmacodynamic effect
verapamil	CYP3A4/P-gp inhibitor	CYP3A4/P-gp inhibitor	CYP3A4/P-gp inhibitor	CYP3A4/P-gp inhibitor
vinblastine	CYP3A4/P-gp inducer	CYP3A4/P-gp inducer	CYP3A4/P-gp inducer	CYP3A4/P-gp inducer
vorapaxar	Pharmacodynamic effect	Pharmacodynamic effect	Pharmacodynamic effect	Pharmacodynamic effect
voriconazole	CYP3A4/P-gp inhibitor	CYP3A4/P-gp inhibitor	CYP3A4/P-gp inhibitor	CYP3A4/P-gp inhibitor
voxilaprevir	NA	CYP3A4/P-gp inhibitor	NA	NA

Supplementary table 4. Potential NOAC-drug interactions at index, in 244,597 patients with newly initiated NOAC treatment during 2008 – 2017 in Swedish outpatient care. Values are given as n (%).

	**Apixaban n = 148108**	**Rivaroxaban n = 59276**	**Dabigatran n = 35617**	**Edoxaban n = 1596**
**Cardiovascular drugs**				
Amiodarone	1368 (0.92)	341 (0.58)	207 (0.58)	38 (2.38)
Diltiazem	528 (0.36)	259 (0.44)	195 (0.55)	9 (0.56)
Dronedarone	1135 (0.77)	168 (0.28)	201 (0.56)	129 (8.08)
Propafenone	19 (0.01)	10 (0.02)	20 (0.06)	0 (0)
Verapamil	962 (0.65)	416 (0.7)	250 (0.7)	16 (1)
**Antiepileptics**				
Carbamazepine	525 (0.35)	159 (0.27)	113 (0.32)	19 (1.19)
Levetiracetam	651 (0.44)	248 (0.42)	157 (0.44)	8 (0.5)
Oxcarbazepine	29 (0.02)	13 (0.02)	8 (0.02)	0 (0)
Phenobarbital	48 (0.03)	18 (0.03)	12 (0.03)	1 (0.06)
Phenytoin	118 (0.08)	47 (0.08)	26 (0.07)	4 (0.25)
Primidone	0 (0)	1 (<0.01)	3 (<0.01)	0 (0)
Topiramate	40 (0.03)	22 (0.04)	8 (0.02)	0 (0)
Valproic acid	365 (0.25)	137 (0.23)	61 (0.17)	3 (0.19)
**Antibiotics**				
Clarithromycin	53 (0.04)	19 (0.03)	7 (0.02)	1 (0.06)
Erythromycin	20 (0.01)	12 (0.02)	3 (<0.01)	0 (0)
Rifampicin	56 (0.04)	24 (0.04)	12 (0.03)	5 (0.31)
**Antidepressants**				
Citalopram	5401 (3.65)	1930 (3.26)	1095 (3.07)	53 (3.32)
Escitalopram	1523 (1.03)	597 (1.01)	282 (0.79)	17 (1.07)
Fluoxetine	438 (0.3)	208 (0.35)	115 (0.32)	3 (0.19)
Fluvoxamine	2 (<0.01)	2 (<0.01)	0 (0)	0 (0)
Paroxetine	473 (0.32)	208 (0.35)	120 (0.34)	7 (0.44)
Sertraline	3498 (2.36)	1392 (2.35)	674 (1.89)	24 (1.5)
Clomipramine	180 (0.12)	70 (0.12)	39 (0.11)	5 (0.31)
Duloxetine	653 (0.44)	282 (0.48)	172 (0.48)	12 (0.75)
Venlafaxine	1212 (0.82)	517 (0.87)	264 (0.74)	19 (1.19)
**Antimycotics**				
Fluconazole	159 (0.11)	78 (0.13)	21 (0.06)	5 (0.31)
Itraconazole	2 (<0.01)	0 (0)	4 (0.01)	0 (0)
Ketoconazole	0 (0)	1 (<0.01)	0 (0)	0 (0)
Posaconazole	3 (<0.01)	1 (<0.01)	2 (<0.01)	0 (0)
Voriconazole	1 (<0.01)	1 (<0.01)	1 (<0.01)	0 (0)
**Antineoplastic agents**				
Enzalutamide	50 (0.03)	7 (0.01)	10 (0.03)	3 (0.19)
Abiraterone	22 (0.01)	8 (0.01)	2 (<0.01)	1 (0.06)
Bicalutamide	1033 (0.7)	352 (0.59)	150 (0.42)	13 (0.81)
Tamoxifen	390 (0.26)	192 (0.32)	87 (0.24)	11 (0.69)
**Immunomodulating agents**				

Ciclosporin	76 (0.05)	14 (0.02)	4 (0.01)	2 (0.13)
Dexamethasone	205 (0.14)	49 (0.08)	5 (0.01)	3 (0.19)
Prednisone	162 (0.11)	90 (0.15)	19 (0.05)	2 (0.13)
Tacrolimus	174 (0.12)	42 (0.07)	7 (0.02)	5 (0.31)
Tocilizumab	28 (0.02)	7 (0.01)	4 (0.01)	0 (0)
**Antivirals**				
Atazanavir	1 (<0.01)	2 (<0.01)	0 (0)	0 (0)
Cobicistat	0 (0)	2 (<0.01)	0 (0)	0 (0)
Darunavir	0 (0)	1 (<0.01)	1 (<0.01)	0 (0)
Efavirenz	12 (<0.01)	5 (<0.01)	3 (<0.01)	0 (0)
Glecaprevir	1 (<0.01)	0 (0)	0 (0)	0 (0)
Lopinavir	0 (0)	1 (<0.01)	1 (<0.01)	0 (0)
Pibrentasvir	1 (<0.01)	0 (0)	0 (0)	0 (0)
Ritonavir	0 (0)	1 (<0.01)	1 (<0.01)	0 (0)
Velpatasvir	3 (<0.01)	1 (<0.01)	0 (0)	0 (0)
**Analgesic**				
Tramadol	1520 (1.03)	1351 (2.28)	2428 (6.82)	11 (0.69)
**Antithombotic agents**				
Acetylsalicylic acid	39888 (26.93)	14168 (23.9)	8541 (23.98)	375 (23.5)
Cilostazol	11 (<0.01)	4 (<0.01)	0 (0)	0 (0)
Clopidogrel	7060 (4.77)	2380 (4.02)	1191 (3.34)	69 (4.32)
Dipyridamole	808 (0.55)	211 (0.36)	207 (0.58)	2 (0.13)
Prasugrel	20 (0.01)	12 (0.02)	9 (0.03)	3 (0.19)
Ticagrelor	1202 (0.81)	365 (0.62)	207 (0.58)	17 (1.07)
Ticlopidine	1 (<0.01)	0 (0)	1 (<0.01)	0 (0)
Treprostinil	0 (0)	1 (<0.01)	1 (<0.01)	0 (0)
Dalteparin	3495 (2.36)	1912 (3.23)	535 (1.5)	145 (9.09)
Danaparoid	0 (0)	1 (<0.01)	0 (0)	0 (0)
Enoxaparin	571 (0.39)	236 (0.4)	116 (0.33)	16 (1)
Heparin	3 (<0.01)	2 (<0.01)	0 (0)	0 (0)
Tinzaparin	2488 (1.68)	1026 (1.73)	279 (0.78)	86 (5.39)
Fondaparinux	8 (<0.01)	7 (0.01)	3 (<0.01)	1 (0.06)
**NSAIDs**				
Celecoxib	1105 (0.75)	154 (0.26)	134 (0.38)	6 (0.38)
Dexibuprofen	120 (0.08)	80 (0.13)	63 (0.18)	1 (0.06)
Diclofenac	2038 (1.38)	1622 (2.74)	1818 (5.1)	7 (0.44)
Etoricoxib	4207 (2.84)	2845 (4.8)	782 (2.2)	6 (0.38)
Ibuprofen	1256 (0.85)	666 (1.12)	559 (1.57)	9 (0.56)
Indometacin	1 (<0.01)	3 (<0.01)	8 (0.02)	0 (0)
Ketoprofen	781 (0.53)	424 (0.72)	420 (1.18)	4 (0.25)
Lornoxicam	1 (<0.01)	0 (0)	0 (0)	0 (0)
Meloxicam	59 (0.04)	29 (0.05)	30 (0.08)	0 (0)
Nabumetone	242 (0.16)	87 (0.15)	308 (0.86)	1 (0.06)
Naproxen	3604 (2.43)	1990 (3.36)	1098 (3.08)	19 (1.19)
Piroxicam	13 (<0.01)	6 (0.01)	14 (0.04)	0 (0)

Tenoxicam	53 (0.04)	30 (0.05)	26 (0.07)	0 (0)

Percentages based on the total number of patients dispensed each NOAC.

Supplementary table 5. Number of patients and events during the first 6 months of newly initiated NOAC treatment in 244 597 patients during 2008 – 2017 in Swedish outpatient care.

NOAC	Interacting group			All	Not interacting group	Interacting group
Apixaban	Pharmacodynamic effect	Any severe bleed	n	156280	84871	71409
			Any severe bleed (%)	1616 (1.034)	890 (1.049)	726 (1.017)
		Gastrointestinal bleeding	n	156280	84871	71409
			Gastrointestinal bleeding (%)	617 (0.395)	333 (0.392)	284 (0.398)
		Intracranial bleeding	n	156280	84871	71409
			Intracranial bleeding (%)	276 (0.177)	159 (0.187)	117 (0.164)
	CYP3A4 and/or P-gp inducer	Ischemic stroke/stroke unspecified/TIA	n	156280	153992	2288
			Ischemic stroke/stroke unspecified/TIA (%)	2576 (1.648)	2535 (1.646)	41 (1.792)
		Ischemic stroke	n	156280	153992	2288
			Ischemic stroke (%)	1767 (1.131)	1732 (1.125)	35 (1.53)
		Venous thrombosis	n	156280	153992	2288
			Venous thrombosis (%)	937 (0.6)	918 (0.596)	19 (0.83)
	CYP3A4 and/or P-gp inhibitor	Any severe bleed	n	156280	149049	7231
			Any severe bleed (%)	1930 (1.235)	1827 (1.226)	103 (1.424)
		Gastrointestinal bleeding	n	156280	149049	7231
			Gastrointestinal bleeding (%)	720 (0.461)	676 (0.454)	44 (0.608)
		Intracranial bleeding	n	156280	149049	7231
			Intracranial bleeding (%)	343 (0.219)	334 (0.224)	9 (0.124)
Dabigatran	Pharmacodynamic effect	Any severe bleed	n	38113	19672	18441
			Any severe bleed (%)	472 (1.238)	263 (1.337)	209 (1.133)
		Gastrointestinal bleeding	n	38113	19672	18441
			Gastrointestinal bleeding (%)	216 (0.567)	123 (0.625)	93 (0.504)
		Intracranial bleeding	n	38113	19672	18441
			Intracranial bleeding (%)	88 (0.231)	53 (0.269)	35 (0.19)
	CYP3A4 and/or P-gp inducer	Ischemic stroke/stroke unspecified/TIA	n	38113	37681	432
			Ischemic stroke/stroke unspecified/TIA (%)	899 (2.359)	880 (2.335)	19 (4.398)
		Ischemic stroke	n	38113	37681	432
			Ischemic stroke (%)	680 (1.784)	665 (1.765)	15 (3.472)
		Venous thrombosis	n	38113	37681	432
			Venous thrombosis (%)	139 (0.365)	138 (0.366)	1 (0.231)
	CYP3A4 and/or P-gp inhibitor	Any severe bleed	n	38113	36773	1340
			Any severe bleed (%)	536 (1.406)	523 (1.422)	13 (0.97)
		Gastrointestinal bleeding	n	38113	36773	1340
			Gastrointestinal bleeding (%)	250 (0.656)	243 (0.661)	7 (0.522)
		Intracranial bleeding	n	38113	36773	1340
			Intracranial bleeding (%)	95 (0.249)	94 (0.256)	1 (0.075)
Edoxaban	Pharmacodynamic effect	Any severe bleed	n	2486	968	1518
			Any severe bleed (%)	8 (0.322)	0 (0)	8 (0.527)
		Gastrointestinal bleeding	n	2486	968	1518

			Gastrointestinal bleeding (%)	3 (0.121)	0 (0)	3 (0.198)
		Intracranial bleeding	n	2486	968	1518
			Intracranial bleeding (%)	0 (0)	0 (0)	0 (0)
	CYP3A4 and/or P-gp inducer	Ischemic stroke/stroke unspecified/TIA	n	2486	2459	27
			Ischemic stroke/stroke unspecified/TIA (%)	2 (0.08)	2 (0.081)	0 (0)
		Ischemic stroke	n	2486	2459	27
			Ischemic stroke (%)	1 (0.04)	1 (0.041)	0 (0)
		Venous thrombosis	n	2486	2459	27
			Venous thrombosis (%)	4 (0.161)	2 (0.081)	2 (7.407)
	CYP3A4 and/or P-gp inhibitor	Any severe bleed	n	2486	1865	621
			Any severe bleed (%)	10 (0.402)	8 (0.429)	2 (0.322)
		Gastrointestinal bleeding	n	2486	1865	621
			Gastrointestinal bleeding (%)	3 (0.121)	2 (0.107)	1 (0.161)
		Intracranial bleeding	n	2486	1865	621
			Intracranial bleeding (%)	0 (0)	0 (0)	0 (0)
Rivaroxaban	Pharmacodynamic effect	Any severe bleed	n	62811	32650	30161
			Any severe bleed (%)	1047 (1.667)	528 (1.617)	519 (1.721)
		Gastrointestinal bleeding	n	62811	32650	30161
			Gastrointestinal bleeding (%)	409 (0.651)	204 (0.625)	205 (0.68)
		Intracranial bleeding	n	62811	32650	30161
			Intracranial bleeding (%)	139 (0.221)	72 (0.221)	67 (0.222)
	CYP3A4 and/or P-gp inducer	Ischemic stroke/stroke unspecified/TIA	n	62811	61956	855
			Ischemic stroke/stroke unspecified/TIA (%)	862 (1.372)	844 (1.362)	18 (2.105)
		Ischemic stroke	n	62811	61956	855
			Ischemic stroke (%)	580 (0.923)	568 (0.917)	12 (1.404)
		Venous thrombosis	n	62811	61956	855
			Venous thrombosis (%)	1380 (2.197)	1365 (2.203)	15 (1.754)
	CYP3A4 and/or P-gp inhibitor	Any severe bleed	n	62811	60684	2127
			Any severe bleed (%)	1207 (1.922)	1157 (1.907)	50 (2.351)
		Gastrointestinal bleeding	n	62811	60684	2127
			Gastrointestinal bleeding (%)	471 (0.75)	447 (0.737)	24 (1.128)
		Intracranial bleeding	n	62811	60684	2127
			Intracranial bleeding (%)	163 (0.26)	160 (0.264)	3 (0.141)

Values are given as n (%), percentages based on the total number of patients in each group.
